# PHYSICAL ACTIVITY FRAGMENTATION AND FALLS IN OLDER ADULTS

**DOI:** 10.1093/geroni/igae098.3491

**Published:** 2024-12-31

**Authors:** Braden Popelsky, Kelley Pettee Gabriel, Erin Dooley, Kelly Ylitalo

**Affiliations:** Baylor University, Bryan, Texas, United States; University of Alabama at Birmingham, Birmingham, Alabama, United States; University of Alabama at Birmingham, Birmingham, Alabama, United States; Baylor University, Waco, Texas, United States

## Abstract

Physical activity (PA) may be an important fall prevention strategy. Current PA guidelines emphasize total PA dose, but daily patterning of PA is underappreciated. With aging, PA bouts become less frequent and shorter in duration (i.e., more fragmented). PA fragmentation may be an indicator of fall risk, but the relationship is not well understood. This study examined daily PA patterns and fall risk in a sample of older adults from the National Health and Aging Trends Study (NHATS; (n=685, 54.3% female, 61.5% aged 70–79 years). Wrist-worn accelerometry from Round 11 (2021) measured PA and surveys from Round 12 (2022) measured falls. PA variables were categorized into tertiles and incident falls were defined as ≥1 self-reported fall in the year following the PA assessment. A modified Poisson approach was used to estimate the relative risk of total PA and PA fragmentation with falls. Overall, 40.0% reported ≥1 incident fall. After adjustment for sociodemographic and health characteristics, those in the highest tertile of total PA accumulation had lower fall risk (aRR=0.74; 95% CI: 0.57, 0.95) and those in the highest tertile of PA fragmentation had increased fall risk (aRR=1.33; 95% CI: 1.02, 1.74) compared to those in the lowest tertiles, respectively. Models were attenuated after the adjustment for physical functioning. PA fragmentation may identify fall risk in older adults. More work is needed to understand the complex relationship between PA and physical functioning across the life-course.

